# Unraveling Etiological Indications and Therapeutic Implications of Familial Cerebral Cavernous Malformations in the Dawn of Gene Therapy for Monogenic Conditions

**DOI:** 10.2174/0115665232386151250812105845

**Published:** 2025-08-19

**Authors:** Ke Ma, Moksada Regmi, Shikun Liu, Ying Xiong, Yingjie Wang, Weihai Liu, Yuwei Dai, Guozhong Lin, Jun Yang, Chenlong Yang

**Affiliations:** 1 Department of Neurosurgery, State Key Laboratory of Vascular Homeostasis and Remodeling, Peking University Third Hospital, Beijing 100191, China;; 2 Center for Precision Neurosurgery and Oncology of Peking University Health Science Center, Peking University, Beijing 100191, China;; 3 Peking University Health Science Center, Beijing 100191, China;; 4 Center for Oculocranial Pressure Instability Disorders (COPID), Henan Academy of Innovations in Medical Science (AIMS), Zhengzhou 450003, China

**Keywords:** Cerebral cavernous malformations, gene therapy, monogenic disease, CRISPR Cas9, base editing, central nervous system

## Abstract

Cerebral Cavernous Malformations (CCMs) are vascular anomalies in the central nervous system that arise from both genetic and non-genetic factors, and can cause hemorrhage, seizures, and neurological deficits. Approximately 80% of CCMs are sporadic, while 20% are Familial (FCCMs), an autosomal dominant, monogenic disorder characterized by multiple lesions and severe clinical manifestations. Over the past three decades, linkage analyses have identified *KRIT1/CCM1*, *MGC4607/CCM2*, and *PDCD10/CCM3* as major pathogenic genes in FCCMs. However, existing surgical and pharmacological treatments have not adequately prevented disease progression, underscoring the need for more effective strategies. Recent advancements in gene editing tools and delivery systems have transformed gene therapy from a laboratory concept to a clinical reality, offering renewed hope for FCCM patients. Given the multifactorial nature, complexity, and neurological comorbidities of FCCMs, exploring non-surgical gene therapies provides a promising approach for addressing these cerebrovascular lesions. This review summarizes the latest progress in gene editing for FCCMs and examines its therapeutic potential, while acknowledging both the promising benefits and the remaining uncertainties in this evolving field.

## 1 INTRODUCTION

Cerebral Cavernous Malformations (CCMs), also known as cavernomas, are characterized by multiple lesions in the Central Nervous System (CNS), consisting of collections of structurally abnormal, slow-flowing capillaries [1, 2]. These lesions are more commonly found in the supratentorial region than in the infratentorial region [2]. The majority of CCM cases, referred to as sporadic CCMs, involve a single lesion and are typically asymptomatic and non-hereditary. In contrast, Familial Cerebral Cavernous Malformations (FCCMs) are a monogenic disorder characterized by multiple lesions and more severe clinical symptoms. The population incidence of FCCMs is estimated to be between 1 in 3,300 and 1 in 10,000 [3]. Affected parents have a 50% chance of passing the genetic mutation to their offspring, leading to significant familial clustering of FCCMs. Consequently, each generation within an affected family has a high probability of carrying pathogenic genes [4]. Despite this classic autosomal dominant inheritance pattern, some studies hint at the involvement of secondary genetic or epigenetic modifiers that may influence the penetrance and severity of FCCMs. Future work dissecting such modifier loci could be pivotal in understanding cases where the known causal genes do not fully explain the clinical outcome.

## 2 METHODOLOGY

This narrative review summarizes existing research on Familial Cerebral Cavernous Malformations (FCCM) and gene therapy without employing systematic review or meta-analysis protocols. Literature was retrieved from PubMed and Web of Science up to 2025 using a combination of MeSH and free-text terms, including “FCCM,” “Gene Therapy,” “CRISPR,” “Base Editing,” and “Prime Editing,” with Boolean operators (AND/OR) to structure the search. A snowballing method was also used to identify additional relevant studies from reference lists. Included studies focused on FCCM pathogenesis, gene editing tools (*e.g*., CRISPR-Cas9, base editing, prime editing), delivery systems (*e.g*., AAV, LNP), and preclinical or translational research. Priority was given to recent peer-reviewed publications from the past five years, with foundational studies on KRIT1, CCM2, and PDCD10 genes included where relevant. Exclusion criteria comprised studies lacking relevance, non-English publications, duplicates, and those without primary data. The literature was organized according to the sequence: FCCM pathogenesis, gene editing technologies, delivery systems, and clinical considerations. Where findings were inconsistent, comparisons were made based on methodological differences, sample sizes, and experimental models. The review emphasizes key challenges in FCCM gene therapy—such as blood-brain barrier delivery, correction thresholds for mosaic lesions, and immunogenicity—and discusses the potential applicability of newer gene editing tools like glycosylase base editing and RNA editors, such as Cas13a, in addressing these issues.

## 3 PATHOGENIC GENES OF FCCMS

**Fig. (1):**
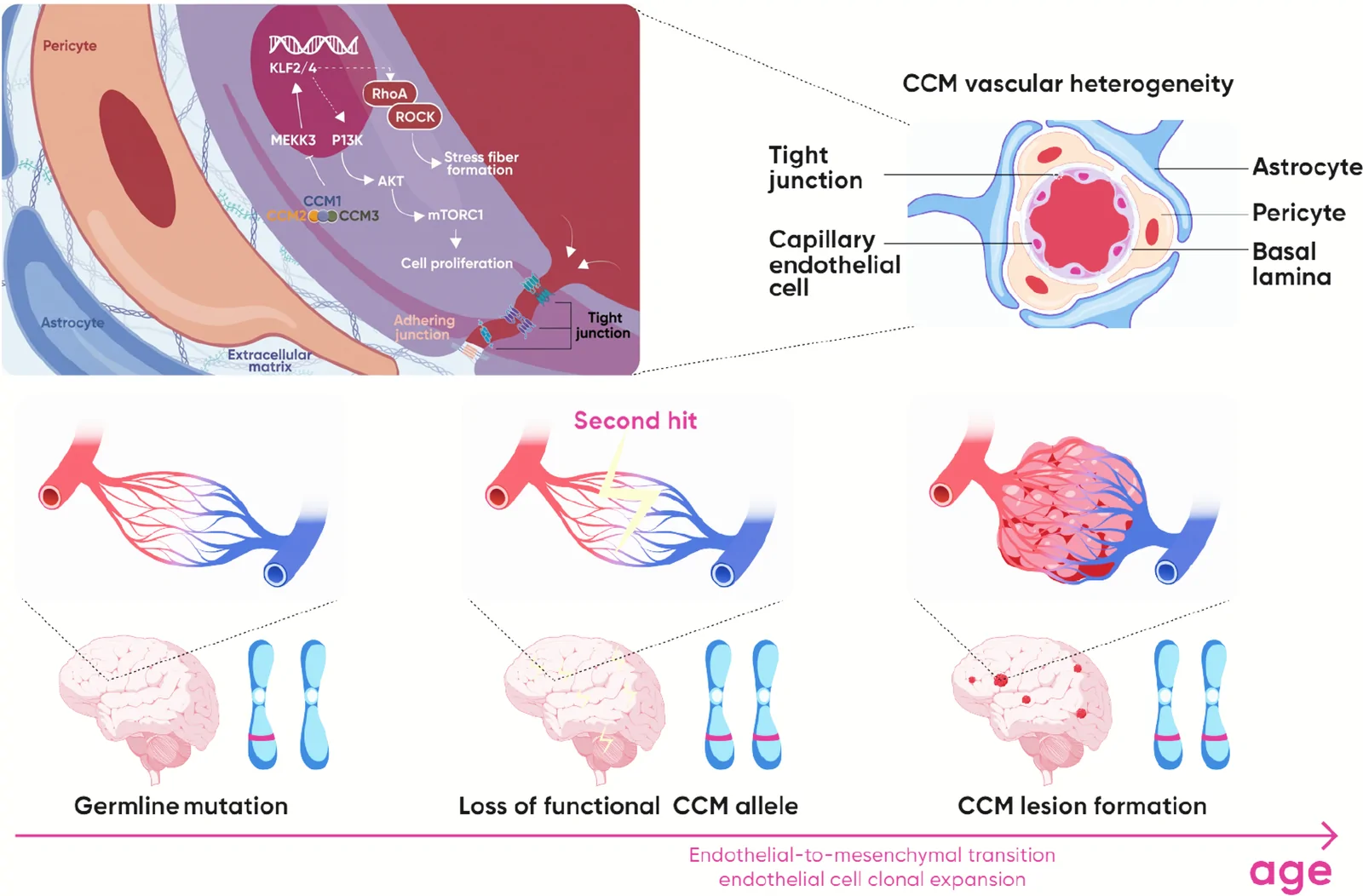
Pathogenic Mechanism of FCCMs. The pathogenesis of FCCMs arises from a two-hit mutation mechanism. The first hit is a germline mutation, while the second hit is a somatic mutation, leading to multifocal lesions. Mutations in CCM genes result in impaired protein expression and function, disrupted cell-cell connections, and increased cellular proliferation, ultimately causing vascular malformations and rupture.

Familial Cerebral Cavernous Malformations (FCCMs) are recognized as an autosomal dominant monogenic genetic disorder, which follow a two-hit mutation model: germline mutation plus a secondary somatic event (Fig. **1**). Through linkage analysis, pathogenic genes associated with FCCMs, including *KRIT1/CCM1*, *MGC4607/CCM2*, and *PDCD10/CCM3*, have been identified [5, 6]. However, these three mutations are not found in more than 5% of FCCM cases [7]. Global gene sequencing studies have identified over one hundred mutation sites within these three chromosomal regions. Significant genetic heterogeneity is observed in the mutation sites across different populations (such as Hispanic, Latino, and Mexican populations), and notable differences exist in the proportion of FCCMs caused by *KRIT1*, *MGC4607*, and *PDCD10* mutations [8]. In the Han population, nearly ten mutation sites have been identified in FCCM families, with the vast majority located within the *KRIT1* gene [9-15]. Mutations in CCM genes lead to loss of protein function, resulting in cellular dysfunction and disease. The most common mutation type is protein truncation, although missense mutations, leading to protein misfolding, are also rare [6]. Notably, a residual subset of FCCM patients (estimated to be 5% or more) remains genetically unexplained, suggesting that large deletions, cryptic regulatory variants, or even newly discovered genes might be implicated. Establishing uniform standards for variant interpretation—*via* functional assays or advanced long-read sequencing—will be essential in narrowing this gap.

Extensive laboratory research spanning 20 years has explored the molecular mechanisms underlying CCM development [16, 17]. The three genes, *KRIT1*, *CCM2*, and *PDCD10*, collectively regulate cell proliferation, network formation, and endothelial growth within a larger signaling pathway. These genes directly interact as part of a heterotrimeric adaptor complex known as the CCM complex, which explains how mutations in three distinct genes can result in the same disease while encoding three non-homologous proteins [18, 19]. Mutations in the CCM genes are believed to impair critical surface interactions between these proteins and numerous other binding partners [6]. The loss of the CCM complex leads to the upregulation of MEKK3 signaling, which subsequently triggers the pathological overexpression of downstream transcription factors, namely KLF2 and KLF4, along with an increase in Rho-associated protein kinase (ROCK) activity. These molecular events contribute to the development of endothelial cell pathology [20]. Notwithstanding the prominence of the MEKK3–KLF2/4 pathway, additional overlaps with the PI3K-mTOR or Wnt–β-catenin cascades may exist, and this cross-talk could be particularly relevant when exploring combination therapies or understanding different clinical courses in patients.

## 4 CLINICAL MANIFESTATIONS AND CELLULAR PATHOLOGY

CCMs can occur at various locations within the CNS, with supratentorial lesions being more prevalent than infratentorial lesions [2]. Pathologically, FCCM lesions do not show significant differences from sporadic CCM. However, familial cases are more likely to affect multiple organs, particularly the brain, retina, skin, vertebrae, liver, and kidneys [21]. Patients with FCCM may experience symptoms such as seizures (40%-70%), focal neurological deficits (25%-50%) without intracranial bleeding, nonspecific headaches (10%-30%), and intracranial hemorrhage (25%-32%), which may be confined to the lesion or extend beyond it. Hemosiderin deposits surrounding individual cavernomas indicate remote leakage caused by abnormal capillaries [7, 22, 23]. Early detection of FCCM is challenging, with a significant proportion of cases (20%-50%) remaining asymptomatic and often being incidentally discovered during head imaging [24]. Nevertheless, as lesions evolve, recurrent micro-hemorrhages may gradually accumulate iron deposits, potentiating both local neurotoxic effects and inflammation. Most non-bleeding lesions are asymptomatic, resulting in a low clinical diagnosis rate. However, when bleeding occurs within a lesion, it can manifest as symptoms such as headaches, seizures, stroke, or neurological dysfunction. Lesions located in the brainstem or critical functional areas can lead to paralysis, aphasia, respiratory disorders, and even death, posing a serious threat to the life and well-being of affected individuals [25]. Significantly, the mosaic structure of CCMs—where not all endothelial cells harbor the primary mutation—adds further complexity to predicting which lesions will become symptomatic. FCCMs are characterized by multiple lesions, an earlier age of onset in children, and relatively severe symptoms. CCMs are the second most common vascular malformation of the CNS, after developmental venous anomalies [2]. Recent studies report a prevalence of CCM ranging from 0.1% to 0.8% [26, 27]. The population-based Scottish Intracranial Vascular Malformation Study (SIVMS) estimated an annual incidence rate of 0.56 per 100,000 individuals aged 16 years and older [28]. Currently, most treatment strategies for CCM focus primarily on the sporadic form, aiming to stabilize the lesion and prevent its growth and bleeding [22]. Unfortunately, a significant proportion of patients still face the challenge of experiencing at least one stroke event during their lifetime [21]. With advancements in neuroimaging technology, it is now possible to accurately assess the number, location, and bleeding stage of CCM lesions using gradient echo sequences or Magnetic Resonance Imaging (MRI). However, the unpredictable nature of bleeding and the lack of effective preventive measures indicate that FCCM patients require lifelong monitoring for the risk of bleeding in their brain lesions.

The exploration of new therapies has faced significant challenges. Clinical trials conducted by a joint team from the University of Chicago and Johns Hopkins University investigated the role of Atorvastatin, a RhoA/ROCK inhibitor, in controlling the progression and bleeding of CCM lesions. However, no effective evidence has been demonstrated so far [29, 30]. In 2021, a collaborative study between the United States and Germany, published in Nature, suggested that CCM development is driven by the functional deletion of the *KRIT1* gene and the activation of the PI3K-mTOR signaling pathway. The study also proposed that Sirolimus, a specific inhibitor of mTOR, could inhibit the progression of CCM lesions. However, clinical trials in this area have not produced ideal results. Researchers at the University of California discovered that Propranolol, a β1 adrenergic receptor antagonist, could improve CCM lesions. Building on this finding, an Italian research team proposed a clinical trial program called “Treat_CCM,” but the latest results published in 2023 were unsuccessful [3, 31, 32]. Such setbacks show the complexity of targeting downstream pathways in CCM biology. Even if a candidate drug shows mechanistic promise in preclinical models, inter-patient heterogeneity and the mosaic nature of CCM lesions can complicate real-world efficacy. Given the current treatment strategies and ongoing research, gene editing tools still hold promise for the treatment and potential cure of FCCM, offering a glimmer of hope for alleviating symptoms.

## 5 GENE EDITING AS A THERAPEUTIC STRATEGY

Familial Cerebral Cavernous Malformation (FCCM) is a monogenic, loss-of-function disorder, making it an ideal target for precision gene repair rather than gene knockout. Contemporary editing platforms fall into two functional classes: disruptive editors, which silence genes, and corrective editors, which restore wild-type sequence. Because FCCM pathogenesis arises from truncated or misspliced CCM proteins, corrective tools—especially Base Editing (BE) and Prime Editing (PE)—are preferred; they can repair single-nucleotide errors without generating double-strand breaks or disruptive indels.

The most frequently mutated locus, *KRIT1*, spans 19 exons; exon 16 encodes Ankyrin repeats, an NPXY motif, and a FERM domain that together anchor RAP1A and ICAP1α, preserving endothelial junctions [33-36]. Similar loss-of-function variants in *CCM2* and *PDCD10* converge on the same pathway. Importantly, FCCM lesions are mosaic, meaning that only a proportion of endothelial cells carry the germline + somatic “second hit.” Modelling studies and preclinical data indicate that correcting as little as 15–30% of these cells can re-establish barrier integrity, dampen ROCK signalling, and halt lesion enlargement. Thus, even partial, high-fidelity repair by BE or PE may deliver clinical benefit while minimizing vector load and off-target risk.

Long-term efficacy depends on whether the chosen platform confers a permanent (DNA-level) or transient (RNA-level) correction. BE and PE write permanent changes and therefore suit one-time or limited-dosing strategies; RNA-guided editors, such as Cas13-ADAR, act only on transcripts and will require redosing, but carry a lower risk of irreversible off-target effects. The sections below compare these options in detail.

### 5.1 *In Vivo vs*. *Ex Vivo* Editing

**Fig. (2):**
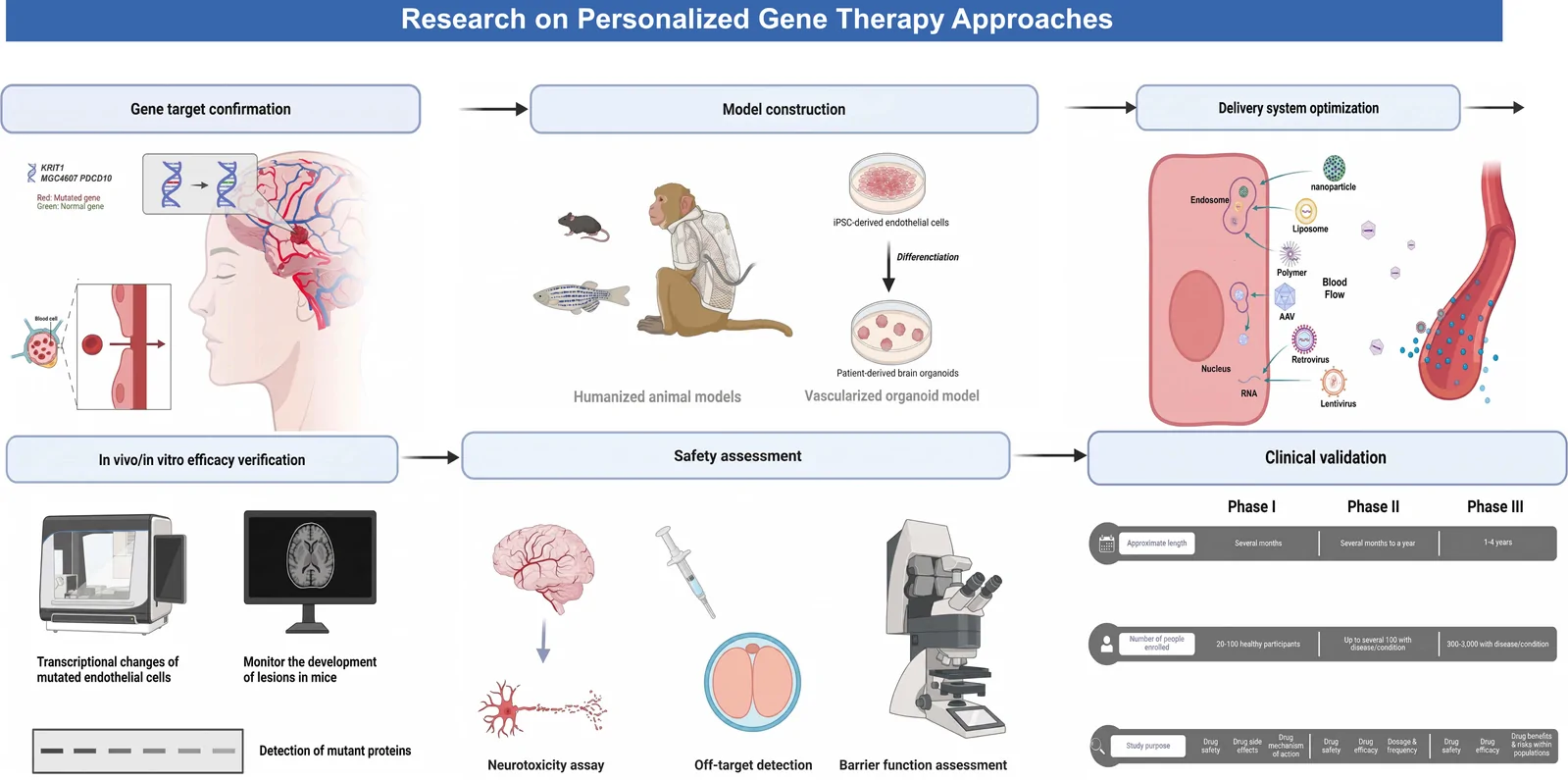
Stepwise Development Pipeline for Personalized Gene Therapies Targeting FCCMs. The process begins with gene target confirmation, focusing on pathogenic variants in KRIT1, CCM2, and PDCD10. Disease models are then constructed using humanized animal models and vascularized brain organoids derived from patient-specific iPSC-endothelial cells. Delivery system optimization involves evaluating various vectors, including nanoparticles, liposomes, AAV, and lentivirus, for CNS targeting and efficient intracellular uptake. Efficacy verification is conducted through transcriptomic profiling, protein detection, and lesion monitoring in preclinical models. Safety assessments include off-target analysis, barrier integrity evaluation, and neurotoxicity testing. The final stage—clinical validation—progresses through Phase I–III trials, which assess safety, dose, and therapeutic benefit across diverse patient cohorts.

Developing FCCM therapies begins with determining whether corrections are made outside the body (*ex vivo*) or directly in the patient (*in vivo*) (Fig. **2**).

*Ex vivo* editing—successfully applied in blood disorders and lymphomas [37-39] —relies on harvesting cells, editing them, and reinfusing them. That workflow is impractical for FCCM: endothelial cells would need to be isolated from multiple CNS lesions, expanded, and surgically re-implanted, all with high immunologic risk [40, 41].

*In vivo* editing delivers CRISPR-based tools (or mRNA/RNP equivalents) straight to the cerebrovasculature [42]. It has already demonstrated clinical benefits in neuromuscular and retinal diseases and, more recently, in a one-shot lipid-nanoparticle therapy for sickle-cell disease [43-45]. For FCCM, the same concept must be adapted to cross the Blood-Brain Barrier (BBB), reach distributed lesions, and limit exposure of healthy vasculature. Permanent editors, such as BE or PE, are attractive here: if even 15-30% of mutant endothelial cells are corrected, junctional integrity can be restored and lesion growth can be arrested, reducing the need for repeat dosing. By contrast, RNA editors offer transient rescue but would require periodic redelivery—an important trade-off when planning lifelong management.

*In vivo* work is technically demanding: vectors must evade immunity, scale to multilesion dosing, and remain effective if neutralizing antibodies emerge with redosing [46]. Nanoparticles or virus-like particles—possibly combined with transient immunosuppression—are under active investigation to meet these criteria. Continued optimization will determine whether durable, one-time BE/PE therapy or repeatable, transcript-level RNA editing (with its inherently lower permanent off-target risk) becomes the frontline strategy for FCCM.

### 5.2 Delivery Strategies

For *in vivo* therapies to succeed in FCCM, delivery vectors must be stable in circulation, resist immune clearance, cross the BBB, and efficiently target affected endothelial cells [40]. This is especially challenging given the architecture of FCCM lesions—clusters of distended, thin-walled capillaries devoid of normal tissue support [2].

Among delivery vehicles, Adeno-Associated Viruses (AAVs) and Lipid Nanoparticles (LNPs) are the most studied. AAV-BI30 has shown strong tropism for CNS endothelium at low doses in mice, making it a leading candidate for CNS-targeted gene editing. However, AAVs have drawbacks, including immunogenicity, limitations in cargo size, and the challenge of redosing due to neutralizing antibodies [47].

Non-viral systems, particularly lipid- or polymer-based nanoparticles, are emerging as attractive alternatives. These carriers are tunable, low in immunogenicity, and capable of encapsulating large editing complexes [48]. Some preclinical work suggests that transient induction of Endothelial-to-Mesenchymal Transition (EMT) may increase nanoparticle uptake, though safety concerns remain regarding EMT induction in the CNS.

Ultimately, carrier systems must achieve sufficient transduction coverage across dispersed lesions while maintaining high selectivity for mutant endothelial cells. Strategies may include ligand-directed nanoparticles or engineered AAVs with improved endothelial tropism. In FCCM, even a small margin of off-target uptake may carry a serious risk, so precise targeting is critical (Table **1**).

## 6 GENE EDITING TOOLS

Gene editing holds promise for correcting the diverse mutations implicated in FCCM, including transition and transversion point mutations, small deletions, and indels. Transition mutations are implicated in disorders like progeria; transversions underlie diseases like sickle cell anemia; and insertions or deletions are the basis of conditions such as Tay-Sachs and cystic fibrosis. In FCCM, the predominant mutation types are loss-of-function variants, particularly protein-truncating mutations in *KRIT1*, *CCM2*, and *PDCD10*. The heterogeneity of FCCM lesions—including their mosaic structure and variable penetrance—places a premium on tools that combine precision, flexibility, and delivery compatibility with the CNS. A comparative overview of major editing tools is provided in Fig. (**3**) and further detailed in Table **2**, summarizing their mutation scope, durability, feasibility of CNS delivery, and suitability for mosaic FCCM lesions.

Tools must also address an important pathophysiological constraint: in FCCM, correcting only a subset of cells within a lesion may be sufficient to stabilize local vasculature. Thus, tools that achieve partial correction with high fidelity can be therapeutically effective, even without universal correction.

Additionally, the permanence of correction varies depending on the tool. DNA-targeting systems, such as CRISPR-Cas9, Base Editors (BEs), and Prime Editors (PEs), induce lasting genomic changes but carry risks of off-target DNA alterations, especially concerning non-regenerating CNS tissue. In contrast, RNA editing systems, including Cas13-ADAR platforms, offer transient, reversible correction of mutant transcripts—safer for sensitive tissues but requiring repeated delivery [49].

### 6.1 CRISPR-Based Platforms

CRISPR-Cas systems have revolutionized gene editing by enabling the precise and programmable manipulation of genetic material. For FCCM, the choice of the CRISPR tool must consider mutation type, editing precision, delivery compatibility with the CNS, and risks associated with off-target effects.

The most widely used system, Cas9, introduces Double-Strand Breaks (DSBs) at specific DNA loci *via* its RuvC and HNH nuclease domains [50-52]. Repair typically occurs through Non-Homologous End Joining (NHEJ), which is fast but error-prone, or Homology-Directed Repair (HDR), which is more precise but less efficient in post-mitotic cells like brain endothelium [53-55].

To improve specificity and reduce genotoxicity, modified enzymes like Cas9 nickase (Cas9n) and Cas12a/b have been developed. These generate single-strand nicks or staggered ends, potentially minimizing off-target activity. Cas12a also recognizes T-rich PAM sequences, broadening the genome’s editable regions. Meanwhile, Cas13 enzymes offer RNA-targeting activity, opening the door for transient transcript-level modulation rather than permanent edits [56, 57].

Despite its utility, the risk of large deletions or chromosomal rearrangements from DSBs remains a serious limitation for CNS applications. Thus, for FCCM, especially in mosaic lesions located in critical brain regions, tools that avoid DSBs—like base editors or prime editors—are increasingly favored. These newer tools offer more control over editing outcomes while still enabling permanent correction, a key therapeutic advantage for inherited cerebrovascular diseases.

### 6.2 Base Editing

Base editing enables precise, single-nucleotide changes without creating DSBs, making it ideal for treating monogenic diseases like FCCM. It relies on a fusion of a catalytically inactive or nickase Cas9 (dCas9/Cas9n) with a cytidine or adenine deaminase, which converts specific bases within a targeted window [58, 59].

Cytidine Base Editors (CBEs) convert C→T (or G→A), while adenine base editors (ABEs) mediate A→G (or T→C). These transitions cover a substantial fraction of known FCCM mutations across *KRIT1*, *CCM2*, and *PDCD10*. Advanced versions, such as BE4max and ABE8e, offer enhanced fidelity, broader PAM compatibility, and reduced off-target rates [58, 60-63].

For FCCM, BE offers several advantages: (i) it can correct pathogenic mutations at the DNA level, offering permanent restoration of gene function; (ii) It is non-disruptive, avoiding the complications of DSBs; and crucially, (iii) it allows correction of mosaic lesions, where editing even a subset of endothelial cells may be sufficient to restore local vascular integrity and suppress inflammatory pathways.

However, base editing has limitations. The editing window is narrow and sequence-and context-dependent. There is a risk of bystander edits at adjacent cytidines or adenines, which must be carefully evaluated, especially in non-regenerating tissues like the brain. Moreover, while BE is generally more efficient than PE, it is limited to transition mutations and cannot address indels or transversions [64].

Nonetheless, for FCCM patients with amenable point mutations, BE offers a compelling balance of efficiency, safety, and therapeutic durability.

### 6.3 Glycosylase-Based Editor

To overcome the limitations of CBEs and ABEs, Glycosylase Base Editors (GBEs) have been developed to introduce non-transition substitutions, such as C-to-G, enabling the correction of a broader range of pathogenic variants [65]. GBEs use a fusion of a cytidine deaminase, a nickase Cas9, and Uracil DNA Glycosylase (UNG) to catalyze multistep base conversion through targeted DNA repair pathways [66].

Unlike standard BEs, GBEs: (i) enable purine↔pyrimidine switching, expanding the types of editable mutations; (ii) offer a narrow, high-fidelity editing window, reducing bystander effects; and (iii) maintain compatibility with dCas9 or Cas9n backbones, aiding delivery and modularity.

Experimental data have shown editing efficiencies up to 72.1% with specificity exceeding 90% in mammalian systems [67]. Integration with transcriptional Pioneer Factors has further expanded editing windows and improved access to otherwise inaccessible genomic loci [68].

For FCCM, GBEs are particularly valuable when a non-transition point mutation is identified. As with BE, correcting even a portion of the mutant endothelial cells may be enough to normalize local vascular signaling, reduce KLF2/4 activation, and prevent lesion growth.

While GBE platforms are still under active development and remain less validated than BEs, their ability to fill gaps in editing scope makes them important candidates in personalized FCCM therapy, especially when used alongside delivery methods tailored to vascular tissues [69-71].

### 6.4 Prime Editing

Prime Editing (PE) represents a versatile and highly programmable gene editing strategy capable of introducing targeted base substitutions, small insertions, and deletions without the need for Double-Strand Breaks (DSBs) or donor DNA templates. It integrates a Cas9 nickase (Cas9n) with an engineered Reverse Transcriptase (RT) and a prime editing guide RNA (pegRNA), which encodes the desired edit and directs it to the target locus [66, 72, 73]. This broad versatility is particularly relevant for FCCM, given the range of known and emerging mutations that might require more than simple transitions.

Unlike base editing, which is limited to transition mutations, PE can theoretically generate all 12 possible base-to-base conversions, as well as targeted indels. This broad editing range makes it especially promising for FCCM, which involves diverse mutations across *KRIT1*, *CCM2*, and *PDCD10*. The system’s flexibility is further enhanced by its ability to introduce edits up to 34 base pairs downstream of the nicking site, increasing its reach across patient-specific mutations [74].

While PE is slightly less efficient than BE in some settings, it carries several advantages: a reduced risk of bystander edits, less susceptibility to sequence context, and a much higher edit-to-indel ratio compared to HDR-based systems [75]. In murine blastocysts, editing frequencies of 1.1%-18.5% have been reported, with precision far exceeding that of Cas9-HDR approaches [76]. While BE offers higher editing efficiencies and lower indel levels, PE provides greater versatility, precision, and targeting scope [77, 78].

In the context of FCCM, prime editing could be particularly useful in lesions with compound or multi-site mutations, or in patients whose mutations lie outside the editable windows of conventional BEs. Moreover, its potential to correct small indels or reintroduce correct splice sites aligns well with the mutation profiles observed in truncating variants.

As with other DNA-targeting methods, PE offers permanent correction, which is desirable for long-term management of FCCM, especially in pediatric cases. However, its effectiveness may vary in low-proliferation cells like brain endothelial cells. In such contexts, the capacity of PE to partially restore function in only a subset of mosaic cells may still suffice to prevent lesion progression, reduce inflammatory signaling, or restore vascular integrity.

Nonetheless, the complexity of PE’s machinery—requiring multiple hybridization steps and a relatively large delivery payload—makes efficient and brain-specific delivery a major translational hurdle. Strategies involving dual-AAV systems, self-replicating RNA, or LNPs will need optimization for CNS use.

### 6.5 RNA Editing: Cas13a

Unlike DNA editing tools, RNA editors offer transient correction by targeting mutant transcripts without altering the genome. This feature is particularly attractive in the CNS, where avoiding permanent off-target damage is a key concern. In FCCM, RNA editing could serve as a safe and reversible intervention, particularly when treating lesions in critical brain regions or when long-term expression of therapeutic proteins is not yet justified.

The Cas13a and Cas13b enzymes recognize specific single-stranded RNAs *via* CRISPR RNA (crRNA) guidance, and can be fused to adenosine deaminases (*e.g*., ADAR2) to enable A-to-I (read as G) conversions [79-81]. Fusion constructs, such as REPAIR and RESCUE, have demonstrated efficient RNA editing in mammalian cells, although early systems were large and challenging to package into AAVs.

Recent innovations have focused on minimizing Cas13 constructs for delivery efficiency. Systems like miniCas13X-ADAR2dd and Cas13bt are small enough to fit into single AAVs while maintaining strong editing activity and low off-target effects [82-84]. These tools have demonstrated A-to-I and C-to-U editing with high fidelity, opening doors for transient, CNS-targeted interventions with minimal risk of permanent genome disruption.

A critical challenge remains the control of collateral cleavage, a known issue with Cas13 enzymes where unintended RNAs are degraded once the enzyme is activated. High-fidelity variants, such as hfCas13d, which have been recently used to treat retinitis pigmentosa, dramatically reduce this effect [85].

In FCCM, Cas13-based RNA editing could be applied in scenarios where short-term suppression of pathogenic transcript expression may delay or reduce lesion development. For example, transcript-level correction of mutated *KRIT1* in high-risk brainstem lesions might provide functional rescue without long-term risk. However, this approach would require repeat dosing, and delivery strategies must be tuned to reach diffuse cerebrovascular targets with high precision [85].

### 6.6 CRISPR-Free Gene Editing Tools

#### 6.6.1 Endogenous RNA Editing

CRISPR-free RNA editing platforms offer a strategy for transient, non-permanent correction of pathogenic transcripts without modifying the genome. These tools recruit native human enzymes, such as ADAR (adenosine deaminase acting on RNA) or APOBEC, to catalyze base conversions in target mRNAs. Because no exogenous protein is introduced, immunogenicity is minimized—a major advantage in CNS applications like FCCM.

One prominent system is LEAPER (Leveraging Endogenous ADAR for Programmable Editing of RNA), first introduced in 2019. LEAPER uses short engineered RNA guides (arRNAs) to recruit endogenous ADARs to convert Adenosine (A) to Inosine (I), which is interpreted as Guanosine (G) during translation. However, early LEAPER constructs exhibited low editing efficiency and occasional bystander effects, especially when arRNA lengths were suboptimal.

To address these limitations, LEAPER 2.0 was developed, utilizing circular arRNAs (circ-arRNA) to resist exonuclease degradation and extend editing duration. Delivered *via* AAV, these guides enabled stable RNA editing in human primary cells and organoids [86, 87].

Several complementary innovations have emerged: (i) CLUSTER, a gRNA optimization platform developed by Thorsten’s group, achieved up to 45% on-target editing in cell culture and 10% in mouse liver with minimal off-target activity; (ii) cadRNA, developed by Prashant Mali’s lab, uses highly stable circular guide RNAs and has demonstrated 53% editing of the *PCSK9* transcript and 12% correction of a nonsense mutation in *IDUA* in mouse models of mucopolysaccharidosis; (iii) In primates, AIMers delivered *via* GalNAc conjugation enabled 50% editing of liver transcripts without bystander effects and persisted for over a month [88-90].

Despite these promising results, translation to FCCM remains challenging. The CNS vasculature presents a more complex environment, where achieving efficient, lesion-specific delivery and sufficient editing coverage across mosaic lesions is more difficult. Furthermore, RNA editing is inherently transient, requiring repeated administration for sustained therapeutic benefit.

Still, endogenous editing holds key advantages: no vector-encoded proteins, lower immune activation, and reversible modulation of gene function. For FCCM, particularly in high-risk or surgically inoperable lesions, transient transcript correction could reduce lesion activity or hemorrhagic risk without risking permanent off-target damage. Future improvements in cell-type-selective delivery and editing efficiency in the CNS endothelium will determine clinical viability [91].

#### 6.6.2 Editing Tools for Nonsense Mutations

Although missense and truncating mutations are more prevalent in FCCM, nonsense mutations that introduce Premature Termination Codons (PTCs) also contribute to pathogenesis in some patients. These can produce non-functional, truncated proteins, making them attractive targets for correction *via* stop codon readthrough technologies.

One such tool is RESTART, a programmable RNA base editor developed by Yi Chengqi’s team. RESTART introduces pseudouridine modifications at PTCs, enabling ribosomes to bypass stop signals and restore translation of full-length proteins [92]. This strategy has been validated in mammalian cells and could be applicable to FCCM variants that truncate *KRIT1*, *CCM2*, or *PDCD10*.

Another approach involves engineered tRNA/tRNA-synthetase pairs (tRNA-aaRS) that suppress stop codons by inserting the correct amino acid during translation. In 2022, Xia Qing’s team demonstrated that AAV-delivered tRNA-aaRS complexes restored dystrophin expression in both murine and patient-derived muscle cells, leading to functional rescue in Duchenne muscular dystrophy models [93].

These strategies do not edit the underlying DNA or RNA, making them non-mutagenic and theoretically repeatable with minimal genomic risk. However, key challenges remain: (i) Specificity: Ensuring readthrough only occurs at pathogenic PTCs without misreading normal stop codons; (ii) Efficiency: Achieving sufficient protein restoration to reverse pathology; (iii) Delivery: Targeting brain endothelial cells, which are dispersed and often embedded in lesion cores.

Given that PTC mutations are relatively rare in FCCM compared to missense variants, the immediate application of these tools is limited. Nonetheless, they provide an additional therapeutic avenue and may complement DNA/RNA editing in future multi-modal strategies. For FCCM cases harboring nonsense mutations, tRNA-based or pseudouridine-mediated readthrough could enable partial protein rescue, potentially mitigating lesion progression without needing full genetic correction.

## 7 OUTLOOK AND CHALLENGES

The localized nature of Cerebral Cavernous Malformations (CCMs) may offer some convenience for drug administration. However, the mosaic structure, multifocal distribution, and delicate location of CCMs in the Central Nervous System (CNS) also create formidable challenges for safety and therapeutic delivery. Although CCM lesions are focal, not all cells harbor the disease-causing mutations. This mosaicism complicates treatment strategies, as partial correction of mutant cells may be insufficient to curb disease progression, and uniform editing across all affected endothelial cells remains difficult. In many disease models, surpassing a minimum threshold of corrected cells can stabilize the pathology; however, for Familial Cerebral Cavernous Malformations (FCCMs), determining this threshold requires advanced *in vivo* models capable of mirroring the human lesion’s heterogeneity.

Equally pressing is the inflammatory dimension of CCM pathology. Loss-of-function mutations in *KRIT1*, CCM2, or *PDCD10* can compromise the endothelium’s antioxidant and anti-inflammatory defenses, allowing neuroinflammatory astrocytes and other immune mediators to exacerbate lesion growth. Links between inflamed CCM endothelium, leukocyte recruitment, and immunothrombosis indicate that neuroinflammation plays a central role in lesion evolution. Thus, a dual therapeutic approach—integrating precise gene editing with anti-inflammatory interventions—may reduce lesion burden and hemorrhagic risk more effectively than gene manipulation alone.

From a technical perspective, FCCM presents a compelling testbed for gene editing, given that it stems from relatively well-defined, monogenic disruptions. Multiple platforms (DNA-targeting edits, RNA-based modifications, and transcriptional modulation) are under active development. Nonetheless, each option must achieve high editing precision within fragile endothelial cells, where off-target events could spur severe neurological consequences. Preclinical models—especially those capable of single-base or prime editing—show promise but must be refined to address delivery efficiency, minimize off-target effects, and ensure the durability of gene correction.

The delivery question remains especially critical. Crossing the Blood-Brain Barrier (BBB) without damaging it requires careful selection or engineering of viral and non-viral vectors. Lipid nanoparticles, exosomes, and novel AAV serotypes with enhanced BBB permeability may be harnessed. Concurrently, strategies for transient BBB disruption might be explored, albeit with caution to avoid inadvertently promoting hemorrhage. Achieving widespread transduction across multiple vascular sites, ensuring adequate dose accumulation, and addressing potential immune neutralization during re-administration all demand continued innovation.

In parallel, personalized therapy becomes increasingly relevant as next-generation sequencing clarifies the genetic heterogeneity of FCCM patients. Accurate diagnostic tools that identify each patient’s mutation profile can guide the choice of gene editor (*e.g*., single-base editors *vs*. prime editors), further elevating treatment efficacy and mitigating off-target concerns. Additionally, combination therapies that stabilize endothelial junctions or dampen inflammatory cascades may synergize with gene correction, offering more comprehensive outcomes and improving patient quality of life.

Despite these technical hurdles, gene editing remains a transformative prospect for FCCM. Advanced editing systems have already generated single-base knock-in models that facilitate in-depth pathophysiological studies, enabling rigorous testing of both efficacy and safety. However, scaling up from bench to bedside necessitates overcoming major regulatory and manufacturing hurdles, ensuring consistent vector production, meeting stringent safety standards, and navigating ethical considerations tied to editing the CNS. Immune responses to repeated dosing, as well as the potential for late-arising genetic alterations, also mandate strict long-term monitoring.

Overall, continued progress in gene editing tools and delivery systems, alongside a deeper understanding of CCM biology, will be pivotal for effective FCCM therapies. By successfully targeting the core genetic deficits underlying CCM lesions, clinicians could one day offer a non-surgical, neuron-sparing alternative that not only lowers hemorrhagic risk but also improves long-term neurological outcomes. Should these strategies succeed, FCCM may stand as a pioneering example for treating other monogenic or multifactorial cerebrovascular diseases, ultimately ushering in a new era of personalized, precision medicine for complex CNS disorders.

## CONCLUSION

This review highlights the promising potential of gene therapy for FCCM, a monogenic disorder driven by mutations in *KRIT1*, *CCM2,* or *PDCD10*. Existing evidence shows that gene-editing tools, such as BE and PE, can precisely correct pathogenic mutations without inducing double-strand breaks, offering a non-surgical strategy to restore vascular endothelial function. Preclinical studies indicate that partial correction of mutant endothelial cells (as low as 15–30%) in mosaic lesions may be sufficient to stabilize vascular barriers and suppress lesion progression, underscoring the feasibility of targeted gene repair.

Delivery systems, particularly AAVs and LNPs, have advanced to enable CNS targeting, though challenges remain in crossing the BBB and achieving uniform transduction across multifocal lesions. The mosaic nature of FCCM lesions and potential immune responses to viral vectors further necessitate optimization of delivery strategies. Additionally, the inflammatory cascade associated with CCM pathogenesis highlights the need for combinatorial approaches that integrate gene editing with anti-inflammatory therapies.

While gene therapy holds transformative promise for FCCM, critical hurdles must be addressed, including defining the minimal threshold of corrected cells required for clinical efficacy, minimizing off-target effects in sensitive CNS tissues, and developing durable, safe delivery methods. Translational success will depend on integrating precision editing with personalized medicine, leveraging patient-specific mutation profiles to tailor therapeutic approaches. Ultimately, overcoming these challenges may establish gene therapy as a cornerstone for treating FCCM and other monogenic cerebrovascular disorders.

## Figures and Tables

**Fig. (3):**
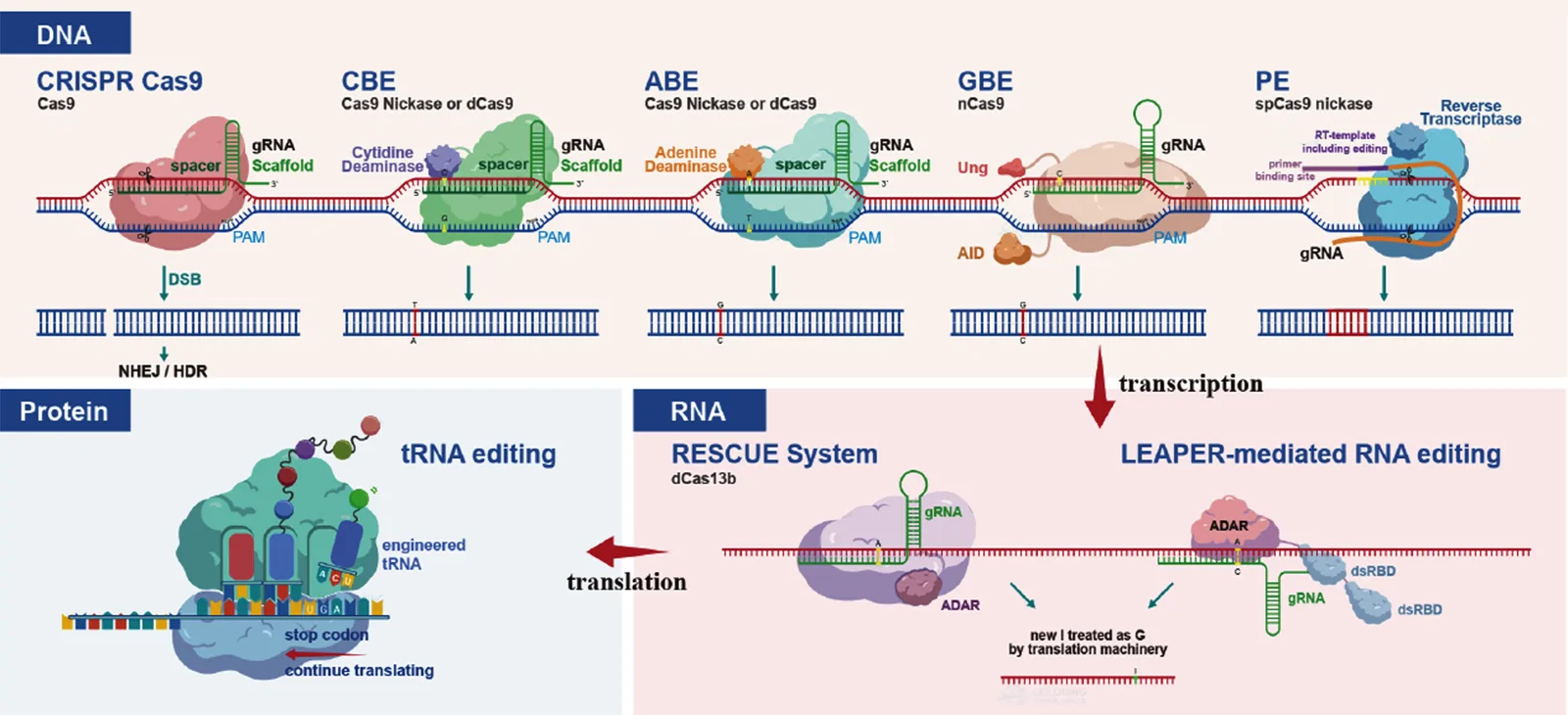
The Working Principles of Different Gene Editing Technologies. FCCMs are associated with the functional deficiency of CCM proteins. Current approaches to address these deficiencies include DNA editing, RNA editing, and protein editing. DNA editing technology is the most advanced, with the potential to cure diseases. Although RNA and protein editing cannot yet achieve curative outcomes, they are rapidly developing and offer higher safety profiles.

**Table 1:** Gene delivery vectors targeting endothelial cells for therapeutic genome editing.

Vector	Mod / Tropism Tag	Cargo (Gene / editor)	Primary Target	Intended Indication	References
**VIRAL VECTORS**					
AAV-BI-hTFR1	7-aa VP1 loop (transferrin-binding)	*NLS-mScarlet; luciferase*	Brain ECs (hTfR)	CNS disorders	[94]
AAV-BR1	Brain-EC-tropic capsid	*HEXA; HEXB*	Cerebral ECs	Tay-Sachs / Sandhoff	[95, 96]
AAV2-GEC	QVLVYRE glomerular peptide	*IdeS*	Glomerular ECs	Inflammatory nephritis	[97]
AAV-GDF11	—	*GDF11*	ECs	Atherosclerosis	[98]
AAV-PHP.eB-NeuroD1	—	*NeuroD1*	ECs	Ischaemic stroke	[99]
AAV9-sFLT1	Soluble FLT1 ectodomain	—	Vascular ECs	Brain AVM	[100]
AAV-BI30	NNSTRGG brain-EC peptide	*NLS-GFP; luc*	Brain microvascular ECs	Endothelial dysfunction	[47]
AAV-PHP.V1	TALKPFL brain-EC peptide	*Cre*	Brain microvascular ECs	—	[101]
AAV2/9-NDVRAVS	NDVRAVS EC-binding peptide	*eGFP*	ECs (HSPG)	—	[102]
rAAV2-ESGHGYF	ESGHGYF lung-EC peptide	*Luc; GFP*	Pulmonary ECs	—	[103]
rAAV2/2[QuadYF-TV]-EC5	Heptameric EC motif	*Mutant rep-cap*	Retinal ECs	Diabetic retinopathy / AMD	[104]
Hybrid AAV (ETP)	Endothelium-targeting peptide	*VWF*	HUVECs	von Willebrand disease	[105]
AAV1-pICAM2-SpCas9	ICAM2 promoter restricts Cas9	*SpCas9*	Retinal ECs	Retinopathy	[106]
Oncolytic AdV	CD40L trimer	*CD40L-IZ*	HUVECs (CD40)	Pancreatic cancer vessels	[107]
AdV-VEGF-DΔNΔC	Truncated VEGF-D insert	*VEGF-DΔNΔC*	HUVECs (VEGFR2/3)	Placental insufficiency	[108]
rFVIII-LV	—	*ABE / PE tools*	BOECs	Haemophilia A	[109]
VE-cadherin LV	VE-cadherin promoter	*VEGFR2 shRNA*	Retinal ECs	Retinopathy	[110]
GP64-LV	Bac-GP64 + CD47 cloak	*FVIII*	Liver ECs	Haemophilia A	[111]
LV.VEC	miRT-shielded FVIII	*FVIII*	LSEC	Haemophilia A	[112]
**NON-VIRAL VECTORS**					
CaP nanoparticle	Calcium-phosphate core	*dCas9-Sirt1 RNP*	Nasal epithelium (MCT1)	Ischaemic stroke	[113]
PEI copolymer NP	P(MMD-co-LA)-g-PEI	*eGFP; ZNF580 plasmid*	Generic ECs	—	[114]
mPEG-PLGA-PEI NP	CREDVW homing peptide	*eGFP; ZNF580 plasmid*	ECs	—	[115]
PEG-PLGA NP	Brain-EC-target polymer	*Cas9 + gRNA (CDH5 promoter)*	Vascular ECs	Cerebrovascular disease	[116]
G2CNN-AAV9‡	G2 dendrimer + EC peptides	*Cre*	Microvascular ECs	Atherosclerosis models	[117]
CDG2-cRGD plasmid[^120]	α_vβ_3-binding cRGD	*VEGF*	HUVECs (α_vβ_3)	Post-injury intimal thickening	[118]

**Note:** ‡ An AAV capsid coated with a synthetic dendrimer. **Abbreviations:** ABE – adenine base editor; AdV – adenovirus; AMD – age-related macular degeneration; AVM – arteriovenous malformation; BOEC – blood-outgrowth endothelial cell; CaP – calcium phosphate; EC – endothelial cell; HSPG – heparan-sulfate proteoglycan; HUVEC – human umbilical-vein EC; LSEC – liver sinusoidal EC; LV – lentiviral vector; NP – nanoparticle; PE – prime editor; RNP – ribonucleoprotein.

**Table 2:** Comparative analysis of gene-editing platforms for FCCM.

Platform	Mutation Types Addressed	Precision	Delivery Feasibility	Permanence	Suitability for Mosaic Lesions	Best use Case in FCCM
**CRISPR-Cas9 (DSB)**	All (including large indels or insertions)	Moderate (DSBs → NHEJ or HDR)	Challenging due to size and off-target risk	Permanent	Risk of genotoxicity; not ideal in CNS	Large insertions or knock-ins; limited CNS utility due to genotoxic risk.
**BE**	Transition mutations (C→T, A→G)	High (no DSBs, narrow window)	High, especially with AAV or LNP	Permanent	Excellent: partial correction can stabilize lesions	Long-term lesion stabilization; effective when ≥15–20% of mutant endothelial cells are corrected.
**PE**	All point mutations, small indels	Very high (programmable pegRNA)	Moderate–high, delivery is still a challenge	Permanent	Excellent: can target diverse mutations	Precise repair of complex or rare variants; ideal when multiple or compound mutations exist.
**Cas13 / ADAR Fusion**	A→I (RNA level only)	High (transcript-level)	High; packaging improving with smaller Cas13s	Transient	Useful for dynamic or acute modulation	Rapid, reversible transcript correction; ideal for high-risk lesions or early intervention in pediatric cases.
**Endogenous RNA Editors**	A→I (*via* native ADARs, no protein delivery)	Moderate (depends on ADAR levels)	Very high (no foreign protein)	Transient	Lower efficiency but low immunogenicity	Allele-specific silencing with minimal immune risk; suited for long-term modulation with repeated low-dose delivery.

**Abbreviations:** BE – base editor; PE – prime editor; DSB – double-strand break; ADAR – adenosine deaminase acting on RNA; CNS – central nervous system; FCCM – familial cerebral cavernous malformation. “Genome altered?” refers to whether permanent DNA changes occur. “Best use-case” describes the clinical niche where each tool is most effective, particularly considering mosaic correction thresholds.

## LIST OF ABBREVIATIONS

ABE: Adenine Base Editor
AA: Amino Acid
ADAR: Adenosine Deaminase Acting on RNA
AAV: Adeno-Associated Virus
AIMers: Antisense-Interfering Molecules
ANK: Ankyrin
APOBEC: Apolipoprotein B mRNA Editing Catalytic Polypeptide
AVM: Arteriovenous Malformation
BBB: Blood-Brain Barrier
BE: Base Editing
CaP: Calcium Phosphate
Cas9n: Cas9 Nickase
CBE: Cytidine Base Editor
CNS: Central Nervous System
crRNA: CRISPR RNA
DSB: Double-Strand Break
EMT: Endothelial-to-Mesenchymal Transition
FCCM: Familial Cerebral Cavernous Malformation
FERM: Four-point-one, Ezrin, Radixin, Moesin Domain
HDR: Homology-Directed Repair
HF: High Fidelity
HSPG: Heparan Sulfate Proteoglycan
IDUA: Iduronidase Alpha-L
ICAP1α: Integrin Cytoplasmic Domain-Associated Protein-1 Alpha
Indel: Insertion or Deletion
KRIT1: Krev-1 Interaction Trapped-1
LEAPER: Leveraging Endogenous ADAR for Programmable Editing of RNA
LNP: Lipid Nanoparticle
LV: Lentiviral Vector
MAP3K3: Mitogen-Activated Protein Kinase Kinase Kinase 3
MRI: Magnetic Resonance Imaging
mRNA: Messenger RNA
NBE: Next-Generation Base Editor
NHEJ: Non-Homologous End Joining
NP: Nanoparticle
NPXY: Asn-Pro-X-Tyr Motif
pamRNA: Prime Editing gRNA Scaffold
PBS: Primer Binding Site
PCSK9: Proprotein Convertase Subtilisin/Kexin Type 9
PDCD10: Programmed Cell Death 10
PE: Prime Editing
pegRNA: Prime Editing Guide RNA
PTC: Premature Termination Codon
RAP1A: Ras-Associated Protein 1A
REPAIR: RNA Editing for Programmable A-to-I Replacement
RESCUE: RNA Editing for Specific C-to-U Exchange
RHOA: Ras Homolog Family Member A
RNP: Ribonucleoprotein
ROCK: Rho-Associated Protein Kinase
RT: Reverse Transcriptase
sgRNA: Single Guide RNA
siRNA: Small Interfering RNA
tRNA: Transfer RNA
UNG: Uracil DNA Glycosylase
ZFNs: Zinc Finger Nucleases
